# Immunoglobulins associated with human tumours in vivo: IgG concentrations in eluates of colonic carcinomas.

**DOI:** 10.1038/bjc.1980.272

**Published:** 1980-10

**Authors:** J. M. MacSween, S. L. Eastwood

## Abstract

The concentrations of IgG in acidic and 3MKCl eluates of resected colonic carcinomas and adjacent normal tissue were determined by radioimmunoassay. The mean concentration of IgG was significantly higher in both acidic and KCl eluates of primary Dukes Stage C tumours than Dukes Stage A tumours. These results provide direct evidence for increased fixation of IgG in vivo by human colonic cancers which had metastasized. Our results also raise the possibility that IgG is more tightly bound to the Stage C tumours. It is likely that this tumour-associated Ig represents antibody to tumour antigens or antigen-antibody complexes, bound to Fc receptors in the tumour.


*Br. J. Cancer* (1980) **42**, 503

### IMMUNOGLOBULINS ASSOCIATED WITH HUMAN TUMOURS *IN VIVO*: IgG CONCENTRATIONS IN ELUATES OF COLONIC CARCINOMAS

J. M. MacSWEEN and S. L. EASTWOOD

*From the Department of Medicine, Dalhousie University and Camp Hill Hospital, Halifax, Nova Scotia, Canada B3H 3G2*

Received 15 January 1980 Accepted 27 June 1980

**Summary.—The concentrations of IgG in acidic and 3MKCl eluates of resected colonic carcinomas and adjacent normal tissue were determined by radioimmunoassay. The mean concentration of IgG was significantly higher in both acidic and KCl eluates of primary Dukes Stage C tumours than Dukes Stage A tumours. These results provide direct evidence for increased fixation of IgG *in vivo* by human colonic cancers which had metastasized. Our results also raise the possibility that IgG is more tightly bound to the Stage C tumours. It is likely that this tumour-associated Ig represents antibody to tumour antigens or antigen–antibody complexes, bound to Fc receptors in the tumour.**

Many observations attest to the immunogenic potential of malignant tumours in experimental animals and man (Old & Boyse, 1966; Perlmann *et al.*, 1977). The products of such immune responses, both antibodies and sensitized lymphocytes, seem capable of damaging tumour cells in defined experimental conditions (Hellstrom *et al.*, 1971; Scornik & Klein, 1978). However, the significance of such responses to host resistance to tumour growth *in vivo* is still unclear, in view of the potential of immune reactants to enhance tumour growth (Prehn, 1972; Shearer *et al.*, 1973) and recent observations suggesting immunological recognition of autologous non-malignant cells (Perlmann *et al.*, 1977; Weksler *et al.*, 1978).

Previous studies have demonstrated that IgG is associated with solid tumours (Witz, 1977), that a proportion of these IgG molecules are antibodies against tumour antigens with cytotoxic potential (Ran *et al.*, 1976) and that such antibodies can be recovered in acidic eluates (Ehrlich & Witz, 1979; Moav & Witz, 1978). Other reports have demonstrated that tumour antigens can be eluted from cell membranes with 3M KCl (Meltzer *et al.*, 1971; Pincus, 1976). While these immune reactants seem important in influencing tumour growth, most of the information which has been used to develop our concepts of tumour-host interactions come from experiments *in vitro* under defined conditions, or from experimental tumours in animals.

Data published by Izsak *et al.* (1974) suggest that human tumours with the greatest malignant potential tend to bind more $^{125}$I anti-IgG than less malignant tumours. Tumour-associated immunoglobulins can influence tumour behaviour in various ways, but initially it would seem appropriate to directly quantitate immunoglobulins bound to human tumours *in vivo* and to correlate this with evidence of tumour progression *in vivo*. The present study was undertaken to (a) compare the concentration of IgG in eluates of localized and disseminated tumours; (b) determine

Correspondence to: Dr J. M. MacSween, Research Building No. 7, Camp Hill Hospital, 1763 Robie Street, Halifax, Nova Scotia, Canada B3H 3G2.

whether KCl eluates of cells previously exposed to acidic buffers contained substantial amounts of IgG, presumably as tightly bound complexes; (c) compare the concentration of IgG in eluates of tumour tissue and corresponding normal tissue.

### MATERIALS AND METHODS

Tissues used in these studies were obtained at the time of surgery from unselected patients with carcinomas of the colon. Blocks of tissue were obtained from freshly resected tumours and from corresponding normal-appearing tissue from the edge of the resected specimens. This consisted of mucosa and adjacent bowel wall. The wet weight of tissue was determined in each case, and was usually 1–5 g. Suspensions were prepared by dicing the tissues with scalpels in a buffer (pH 7·4) containing $Na_2HPO_4$ (0·008M), $NaH_2PO_4$ (0·0015M) and NaCl (0·131M) (PBS). The suspensions were washed ×3 in PBS.

The diced tissues were suspended in 5 ml glycine HCl buffer (0·1M, pH 2·8) for 30 min at room temperature. The tubes were centrifuged at 500 *g* for 10 min, the supernatant fluid was removed and dialysed overnight against PBS. The cells were washed with PBS and suspended in 5 ml 3M KCl overnight at 4°C. The tubes were then centrifuged at 500 *g* and the supernatant fluid dialysed 1 h against 4 changes of PBS.

An additional acidic eluate was obtained from the last 6 tumours to be processed. After exposure to the initial acidic buffer, the tissue was divided into 2. The first aliquot was suspended in 3M KCl as outlined previously. The second aliquot was suspended for a second time in glycine HCl buffer (pH 2·8) for 30 min. After centrifugation, the supernatant fluid was dialysed against PBS and the cells were suspended in 3M KCl in the same way as the other aliquot. The protein concentration in each eluate was determined by the Bio-Rad assay (Bio Rad Laboratories) using bovine serum albumin (BSA) as a standard.

A radioimmunoassay was used to quantitate IgG in the eluates. IgG was purified from the serum of a patient with multiple myeloma with an IgG paraprotein, by ammonium sulphate precipitation and DEAE-cellulose column chromatography. A 10 μg aliquot was labelled to a specific activity of 3·9 μCi/μg with $^{125}I$, using chloramine T as oxidant (Greenwood *et al.*, 1963). The labelled protein was stored in BSA (30 mg/ml) at −20°C. Goat antiserum to human IgG was obtained from Hyland Laboratories, Malton, Ontario. After dialysis against PBS, the proteins in the antiserum were complexed to CNBr-activated sepharose beads (Pharmacia, Dorval, Quebec). Aliquots of ~2 ng of $^{125}I$ IgG in 50μl PBS containing 30 mg/ml BSA, were added to 10μl aliquots of anti-IgG sepharose. A binding curve was determined by using doubling dilutions of anti-IgG sepharose mixed with CNBr sepharose inactivated with 1M ethanolamine (pH 8·0). The anti-IgG sepharose was suspended in the $^{125}I$ IgG for 1 h at 37°C, then washed ×3 in PBS. Radioactivity bound to the sepharose pellets was then determined in a gamma scintillation spectrometer.

Dilutions of anti-IgG sepharose greater than 1:32 substantially reduced the binding of $^{125}I$ IgG, so a 1:32 dilution was used in subsequent inhibition assays. Twenty μl of an IgG standard (Technicon International of Canada Ltd) in duplicate, or triplicate 20μl aliquots of acidic or KCl eluates were added to 10μl aliquots of anti-IgG sepharose. The tubes were held at 37°C for 30 min before adding 50μl $^{125}I$ IgG. After 2 h at 37°C the sepharose was washed ×3 with PBS and the radioactivity in the pellets determined. Eluates with markedly inhibited binding of the $^{125}I$ IgG were reassayed after suitable dilution.

The extent of tumour spread was determined independently by reviewing the records of the operation and the results of pathological examination of the resected specimens and regional lymph nodes. The tumours were classified as proposed by Dukes & Bussey (1958), as localized to the bowel wall (A), local extension to involve the serosa (B) or metastases to regional lymph nodes (C). None of the cases studied had evidence of distant metastases.

### RESULTS

The mean concentrations of protein in the acidic and KCl eluates are shown in Table I. The protein concentrations in the acidic eluates tended to decrease slightly with increased tumour dissemination, whereas the protein in the KCl eluates increased slightly. However, the differences were not statistically significant.

TABLE I.—*Protein concentrations (mean mg/g tissue ± s.d.) in eluates of resected colonic carcinomas*

| Tissue | Dukes' Stage A (n = 6) Acidic eluate | KCl eluate | Dukes' Stage B (n = 12) Acidic eluate | KCl eluate | Dukes' Stage C (n = 9) Acidic eluate | KCl eluate |
|---|---|---|---|---|---|---|
| Tumour | 2·21 ± 0·82 | 0·84 ± 0·32 | 1·60 ± 0·30 | 1·28 ± 0·27 | 0·82 ± 0·09 | 1·35 ± 0·37 |
| Normal | 1·69 ± 0·49 | 0·44 ± 0·08 | 1·54 ± 0·23 | 0·84 ± 0·20 | 1·33 ± 0·26 | 0·77 ± 0·25 |

TABLE II.—*IgG concentrations in eluates of resected colonic carcinomas*

| | | | | IgG (μg/mg protein) | | | |
|---|---|---|---|---|---|---|---|
| | | | | Tumour tissue | | Normal tissue | |
| Age | Sex | Site* | Differentiation† | Acidic eluate | KCl eluate | Acidic eluate | KCl eluate |
| Stage A | | | | | | | |
| 56 | F | HF | M | 17 | 23 | 55 | 14 |
| 64 | F | R | W | 86 | ND | 30 | ND |
| 71 | F | C | | 2·3 | 26 | 3·6 | 116 |
| 74 | F | AC | | 3·4 | 58 | 10 | 10 |
| 74 | M | SC | M | 6 | 8 | 24 | 14 |
| 77 | M | R | M | 4·2 | 10 | 35 | 24 |
| 80 | M | DC | | 73 | 7 | ND | 6·5 |
| Mean | | | | 27·4 ± 13·7 | 22·0 ± 7·9 | 26·3 ± 7·6 | 30·7 ± 17·1 |
| Stage B | | | | | | | |
| 50 | F | R | M | 117 | 2·7 | 37 | 4 |
| 53 | M | SC | M | 78 | 18 | 16 | 266 |
| 63 | F | R | M | 212 | 15 | 18 | 40 |
| 66 | M | SF | P | 1000 | 400 | 195 | 87 |
| 66 | F | SC | P | 30 | 25 | 96 | 40 |
| 71 | M | SC | M | 12 | 33 | 51 | 33 |
| 71 | M | C | M | 286 | 32 | 6·7 | 8 |
| 72 | F | R | | 3·6 | 4 | 5 | 6 |
| 72 | M | DC | M | 74 | 37 | 37 | 240 |
| 74 | F | C | M | 4·9 | 121 | 106 | 206 |
| 77 | F | C | M | 33 | 50 | 10 | 24 |
| 88 | M | DC | | 509 | 262 | 183 | 100 |
| Mean | | | | 196·6 ± 84·8 | 83·3 ± 35·6 | 63·4 ± 19·4 | 87·8 ± 27·6 |
| Stage C | | | | | | | |
| 59 | F | R | M | 190 | 129 | 162 | 200 |
| 64 | F | SC | M | 133 | 60 | 56 | 22 |
| 67 | F | HF | P | 208 | 112 | 92 | 12 |
| 68 | M | C | P | 80 | 65 | 10 | 17 |
| 73 | F | SC | | 160 | 25 | 9 | 28 |
| 77 | F | C | M | 100 | 215 | 113 | 233 |
| 78 | F | C | P | 265 | 500 | 187 | 33 |
| 86 | F | C | P | 91 | 333 | 25 | 60 |
| 94 | F | SF | M | 560 | 600 | 133 | 11 |
| Mean | | | | 198·5 ± 49·5 | 226·5 ± 69 | 87·4 ± 22·1 | 68·4 ± 28·5 |

* C = caecum; AC = ascending colon; HF = hepatic flexure; SF = splenic flexure; DC = descending colon; SC = sigmoid colon; R = rectum.
† W = well differentiated; M = moderately differentiated; P = poorly differentiated.

The concentrations of IgG expressed as μg/mg protein are shown in Table II. The values in both acidic and KCl eluates from the tumour and normal tissues are shown for each patient. The patients are divided into 3 groups on the basis of Dukes' staging. They are listed by increasing age, and the mean and standard errors for the IgG concentrations in the acidic eluates of the Stage C tumours were significantly higher than the Stage A tumours, by Student's unpaired $t$ test ($P < 0{\cdot}02$).

FIGURE.—Mean IgG concentrations in eluates of resected colonic carcinomas. T = tumour, N = normal.

The mean concentration of IgG in the KCl eluates of Stage C tumours was also significantly higher than in the Stage A tumours ($P < 0{\cdot}05$). The concentrations of IgG in the eluates of normal portions of Stage B and C tumours were slightly higher than in Stage A tumours, but the differences were not significant. The IgG concentrations in the KCl eluates of the Stage C tumours were also significantly greater than in the eluates from the corresponding normal tissue ($P < 0{\cdot}05$). The results are similar when the IgG concentrations were expressed as µg/g tissue. There was no apparent relationship between the IgG concentration and age, sex, site of the primary tumour or degree of differentiation.

It was noted that the mean concentrations of both protein and IgG were lower in the KCl eluates than in the preceding acidic eluates in the A and B tumours, but not in the C tumours. This was further investigated by treating identical aliquots of tissue previously exposed to the acidic buffer with either 3M KCl or a second acidic buffer of glycine HCl. The IgG concentrations in these eluates were then determined and are shown in Table III. In all 4 Stage C tumours, the IgC concentrations in the KCl eluates were higher than in the second acidic eluates. There were no consistent differences in the 2 Stage B tumours nor in the eluates from corresponding normal tissue. As yet, no second acidic eluates have been obtained from Stage A tumours. The concentrations of IgG in the eluates from the tumour tissue were considerably higher than in those from the corresponding normal tissue, but the differences were not statistically significant.

TABLE III.—*IgG concentrations (µg/g tissue) in second eluates of resected colonic carcinomas*

| | Tumour tissue | | Normal tissue | |
|---|---|---|---|---|
| Stage | 2nd acidic eluate | KCl eluate | 2nd acidic eluate | KCl eluate |
| B | 2·7 | 47 | 2 | 10 |
| B | 640 | 478 | ND | ND |
| C | 13·6 | 95 | 15·6 | 26 |
| C | 106 | 159 | 86 | 48 |
| C | 215 | 369 | 2·6 | 5 |
| C | 182 | 636 | 15 | 22 |
| Mean | 193·2 | 297·3 | 24·2 | 22·2 |

### DISCUSSION

Our results show significantly greater concentrations of IgG in both acidic and 3M KCl eluates of primary colonic carcinomas with regional metastases (Dukes' Stage 3) than in similar eluates of localized cancers (Dukes' Stage A). Intermediate levels were found in eluates of tumours with local extension (Dukes' Stage B). These differences did not reflect more general differences in protein concentration, since the mean protein concentration in the acidic eluates of the localized tumours was greater than in those with metastases. There were no significant differences between the different stages in the concentrations of IgG in eluates of corresponding normal tissue, suggesting that the differences in the tumour eluates were related to the neoplastic state.

While many reports reveal the presence of tumour-associated antigens in KCl eluates, the presence of substantial amounts of IgG in these eluates suggests that in many cases tumour components with autoantigenic activity may be in the form of complexes. The mean concentration of IgG in the KCl eluates from the tumours with metastases, unlike the localized tumours, was somewhat higher than in the preceding acidic eluates. Since 3M KCl solubilizes cell-membrane antigens (Pincus, 1976), it would seem capable of dissociating tightly bound antibody from the cells, presumably as complexes. This raises the possibility that IgG is more tightly bound to primary tumours with metastases than to localized tumours, and makes it less likely that it represents irrelevant immunoglobulin. Preliminary comparisons of IgG concentrations in acidic and KCl eluates of identical aliquots of cells showed higher concentrations in the KCl eluates of metastatic tumours, whereas the concentrations in the eluates of the corresponding normal tissue were essentially the same. However, as yet, there are too few localized tumours to determine whether there is a consistent difference between the tumour stages in this respect.

Our results also show generally higher concentrations of IgG in eluates of tumour tissue than in eluates of adjacent normal tissue. The difference was statistically significant in the case of KCl eluates from Class C tumours. This difference presumably represents a more precise definition of tumour-associated immunoglobulins than the total immunoglobulin content of tumours.

Previous reports are consistent with our findings. Anti-tumour antibody or antigen–antibody complexes are capable of blocking lymphocyte-mediated cytotoxicity, and may enhance tumour growth (Witz, 1977). Izsak *et al.* (1974) reported that 9/12 miscellaneous human tumours with high malignant potential bound $^{125}$I-labelled anti-human IgG, compared with 5/13 tumours with low malignant potential. Vanky *et al.* (1975) also found that human tumour cells coated with IgG were less efficient in stimulating autologous lymphocytes than those with little IgG. Our results provide direct evidence for the correlation of tumour-associated immunoglobulins and metastasis in carcinoma of the colon.

The nature of the relationship of tumour-bound Ig to tumour stage remains to be determined. One could postulate that tumours with metastases had been present for a longer time, with progressive accumulation of Ig, since older tumours have been observed to contain more Ig in experimental animals (Witz *et al.*, 1974). However, the concept that tumours with metastases are simply a later stage than localized tumours may be an oversimplification. Experimental tumours vary greatly in their propensity to metastasize and indeed, so do different cells within the same tumour (Fidler & Kripke, 1977). Therefore, it is not unreasonable to postulate that tumour spread and metastasis may be associated with different properties of the tumour cells, different host responses, or both (Sugarbaker, 1979). Our results showing increased IgG in eluates of metastasized tumours could be interpreted in several ways. It is possible that the tumours with metastases have different surface properties leading to increased non-specific trapping of immunoglobulins, though this seems unlikely.

Systematic differences in proteolytic activity in different tumour stages could lead to differential degradation of IgG in the tumours (Keisari & Witz, 1973, 1978) though we detected no proteolytic activity in the eluates. Tumours with metastases may contain a higher concentration of antibodies to tumour antigens because of increased numbers of affinity of antigenic sites, or enhanced humoral immune responses in these patients. It may be relevant that whilst binding of high concentrations of antibody may activate complement and damage tumour cells, binding of antibody without complement activation may stimulate tumour growth (Shearer *et al.*, 1973). Fab fragments of IgG may bind to tumour cells and stimulate tumour growth, having lost the capacity to activate complement (Chard *et al.*, 1967; Witz, 1977). It is possible that the IgG activity detected in our assays represents antigenic fragments rather than intact molecules.

It has recently been shown that tumours contain Fc receptors for IgG which may be on infiltrating host mononuclear cells (Wesenberg, 1978). That a substantial proportion of tumour-associated Ig is associated with Fc receptors is suggested by the observation of an inverse relationship between the amount of IgG on human tumours and their Fc receptor activity, and prolonged washing of the tumours decreased surface Ig and increased Fc-receptor activity (Tönder *et al.*, 1976). Fc receptors have much higher affinity for antigen–antibody complexes or aggregated Ig than for native Ig (Dickler, 1974). Such complexes could form when there is antigen shedding from the tumour, which may be associated with tumour progression (Currie & Alexander, 1974). Further, blocking of cytotoxicity for tumour cells by soluble factors is associated with antigen–antibody complexes rather than with free antigen or antibody (Sjögren *et al.*, 1972). It is also known that activation of suppressor T cells requires binding of antigen-antibody complexes to Fc receptors on these cells (Moretta *et al.*, 1979). It has been suggested that enhancement of tumour growth as well as protection of foetal growth during normal pregnancy occurs through activation of suppressor cells by this mechanism (Gleicher *et al.*, 1979). It has also been demonstrated that 2 murine tumour cell lines with high metastatic activity contained a high percentage of Fc receptor-positive cells, whereas a cell line with low metastatic potential had a low percentage of Fc receptor-positive cells (Schirrmacher & Jacobs, 1979).

These observations, though preliminary, may be relevant to our results demonstrating differential *in vivo* trapping of immunoglobulin by human colonic carcinomas.

The authors wish to thank Dr D. T. Janigan and Dr J. D. Arneaud for assistance in procuring tissue, and to Miss Pat Godin for secretarial assistance.

### REFERENCES

CHARD, T., FRENCH, M. E. & BATCHELOR, J. R. (1967) Enhancement of the C57BL leukemia E.L.4 by Fab fragments of isoantibody. *Transplantation*, **5**, 1266.

CURRIE, G. A. & ALEXANDER, P. (1974) Spontaneous shedding of TSTA by viable sarcoma cells: Its possible role in facilitating metastatic spread. *Br. J. Cancer*, **29**, 72.

DICKLER, H. B. (1974) Studies of the human lymphocyte receptor for heat aggregated or antigen-complexed immunoglobulin. *J. Exp. Med.*, **140**, 508.

DUKES, C. E. & BUSSEY, H. J. R. (1958) The spread of rectal cancer and its effect on prognosis. *Br. J. Cancer*, **12**, 309.

EHRLICH, R. & WITZ, I. P. (1979) The elution of antibodies from viable murine tumor cells. *J. Immunol. Methods*, **26**, 345.

FIDLER, I. J. & KRIPKE, M. L. (1977) Metastasis results from preexisting variant cells within a malignant tumor. *Science*, **197**, 893.

GLEICHER *et al.* (1979) Reference not supplied.

GREENWOOD, F. C., HUNTER, W. M. & GLOVER, J. S. (1963) The preparation of $^{131}$I-labelled human growth hormone of high specific radioactivity. *Biochem. J.*, **89**, 114.

HELLSTRÖM, I., HELLSTRÖM, K. E., SJÖGREN, H. O. & WARNER, G. A. (1971) Demonstration of cell-mediated immunity to human neoplasms of various histological types. *Int. J. Cancer*, **7**, 1.

IZSAK, F. C., BRENNER, H. J., LANDES, E., RAN, M. & WITZ, I. P. (1974) Correlation between clinicopathological features of malignant tumors and cell surface immunoglobulins. *Isr. J. Med. Sci.*, **10**, 642.

KEISARI, Y. & WITZ, I. P. (1973) Degradation of immunoglobulins by lysosomal enzymes of tumors —1. Demonstration of the phenomenon using mouse tumors. *Immunochemistry*, **10**, 565.

KEISARI, Y. & WITZ, I. P. (1978) Degradation of cell bound antibodies by tumor cells from C57BL/6 mice. *J. Natl Cancer Inst.*, **61**, 1135.

MELTZER, M. S., LEONARD, E. J., RAPP, H. J. & BORSOS, T. (1971) Tumor-specific antigen solubilization by hypertonic potassium chloride. *J. Natl Cancer Inst.*, **47**, 703.

MOAV, N. & WITZ, I. P. (1978) Characterization of immunoglobulins eluted from murine tumor cells: Binding patterns of cytotoxic anti-tumor IgG. *J. Immunol. Methods*, **22**, 51.

MORETTA, L., MINGARI, M. C., MORETTA, A. & COOPER, M. D. (1979) Human T lymphocyte subpopulations: studies of the mechanism by which T cells bearing Fc receptors for IgG suppress T-dependent B cell differentiation induced by pokeweed mitogen. *J. Immunol.*, **122**, 984.

OLD, L. J. & BOYSE, E. A. (1966) Specific antigens of tumors and leukemias of experimental animals. *Med. Clin. N. Am.*, **50**, 901.

PERLMANN, P., TROYE, M. & PAPE, G. R. (1977) Cell-mediated immune reactions to human tumors. *Cancer*, **40**, 448.

PINCUS, J. H. (1976) Solubilization of biologically active cell surface antigens. *Contemp. Top. Mol. Immunol.*, **5**, 87.

PREHN, R. T. (1972) The immune reaction as a stimulator of tumor growth. *Science*, **176**, 170.

RAN, M., KLEIN, G. & WITZ, I. P. (1976) Tumor bound immunoglobulins. Evidence for the *in vitro*

coating of tumor cells by potentially cytotoxic anti-tumor antibodies. *Int. J. Cancer*, **17**, 90.

SCHIRRMACHER, V. & JACOBS, W. (1979) Tumor metastases and cell-mediated immunity in a model system in DBA/2 mice. VIII. Expression and shedding of Fc receptors on metastatic tumor cell variants. *J. Supramol. Structure*, **11**, 105.

SCORNIK, J. C. & KLEIN, P. A. (1978) Antibody-dependent lysis of tumor cells *in vivo*. 1. Early lysis of tumor cells. *J. Natl Cancer Inst.*, **61**, 1143.

SHEARER, W. T., PHILPOTT, G. W. & PARKER, C. W. (1973) Stimulation of cells by antibody. *Science*, **182**, 1357.

SJÖGREN, H. O., HELLSTRÖM, I., BANSAL, S. C., WARNER, G. A. & HELLSTRÖM, K. E. (1972) Elution of "blocking factors" from human tumors, capable of abrogating tumor-cell destruction by specifically immune lymphocytes. *Int. J. Cancer*, **9**, 274.

SUGARBAKER, E. V. (1979) Some characteristics of metastasis in man. *Am. J. Pathol.*, **97**, 623.

TÖNDER, O., KRISHNAN, E. C., JEWELL, W. R., MORSE, P. A. & HUMPHREY, L. J. (1976) Tumor Fc receptors and tumor-associated immunoglobulins. *Acta Path. Microbiol. Scand.* [C] **84**, 105.

VANKY, F., TREMPE, G., KLEIN, E. & STJERNSWÄRD, J. (1975) Human tumor–lymphocyte interaction *in vitro*: Blastogenesis correlated to detectable immunoglobulin in the biopsy. *Int. J. Cancer*, **16**, 113.

WEKSLER, M. E., KUNTZ, M. M., BIRNBAUM, G. & INNES, J. B. (1978) Lymphocyte transformation induced by autologous cells. *Fed. Proc.*, **37**, 2370.

WESENBERG, F. (1978) Fc γ receptors and IgG associated with human malignant tumors. *Acta Pathol. Microbiol. Scand.* [C] **86**, 259.

WITZ, I. P. (1977) Tumor-bound immunoglobulins: *In situ* expressions of humoral immunity. *Adv. Cancer Res.*, **25**, 95.

WITZ, I. P., KINAMON, S., RAN, M. & KLEIN, G. (1974) Tumor-bound immunoglobulins. The *in vitro* fixation of radioiodine-labelled anti-immunoglobulin reagents by tumor cells. *Clin. Exp. Immunol.*, **16**, 321.


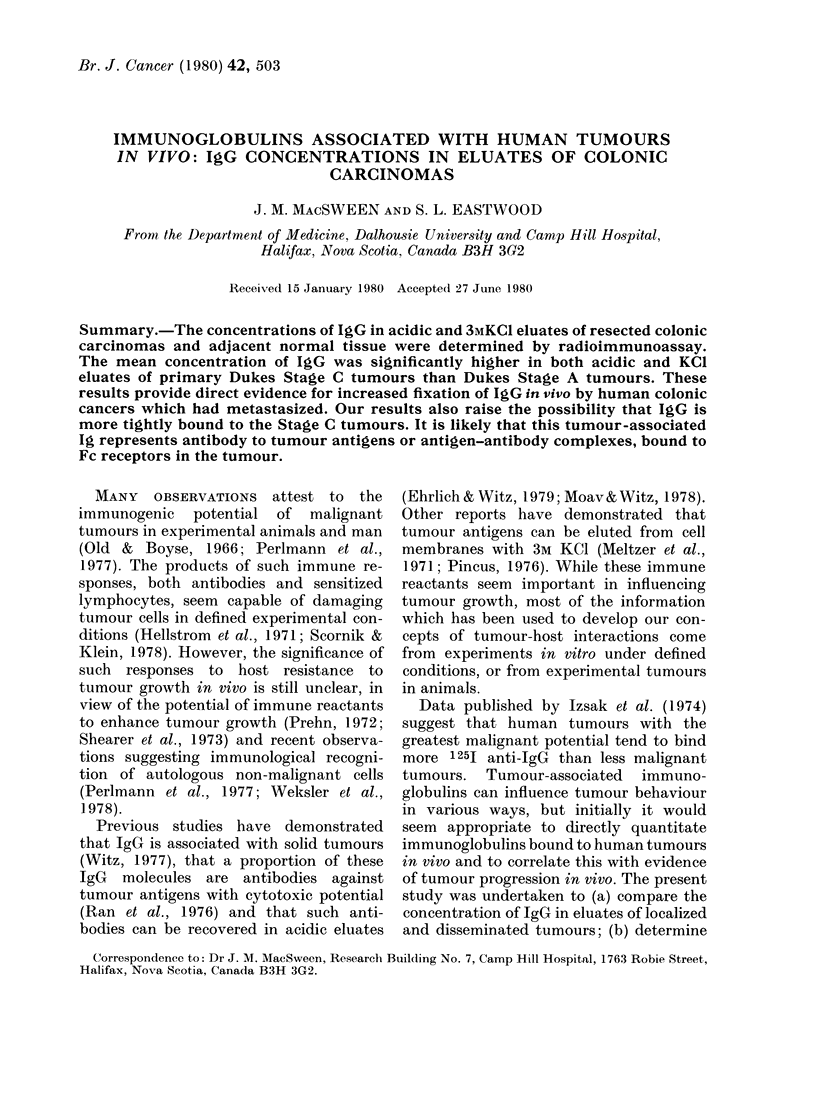

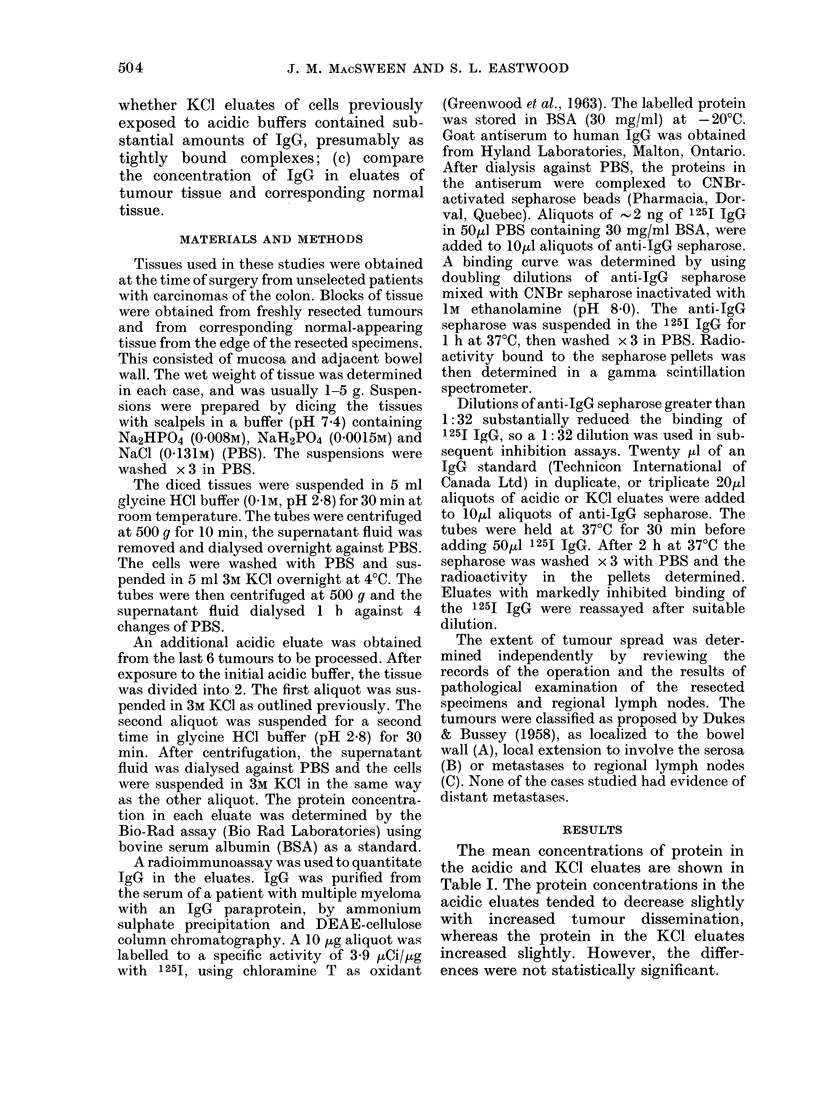

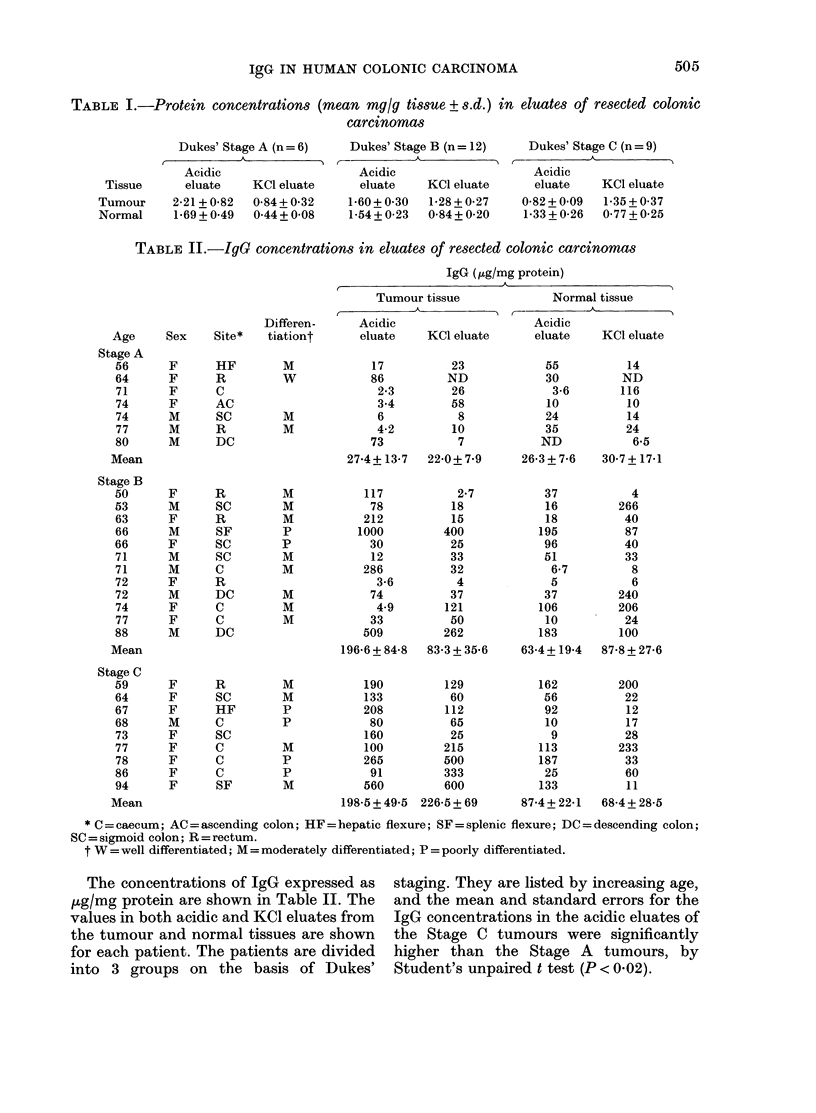

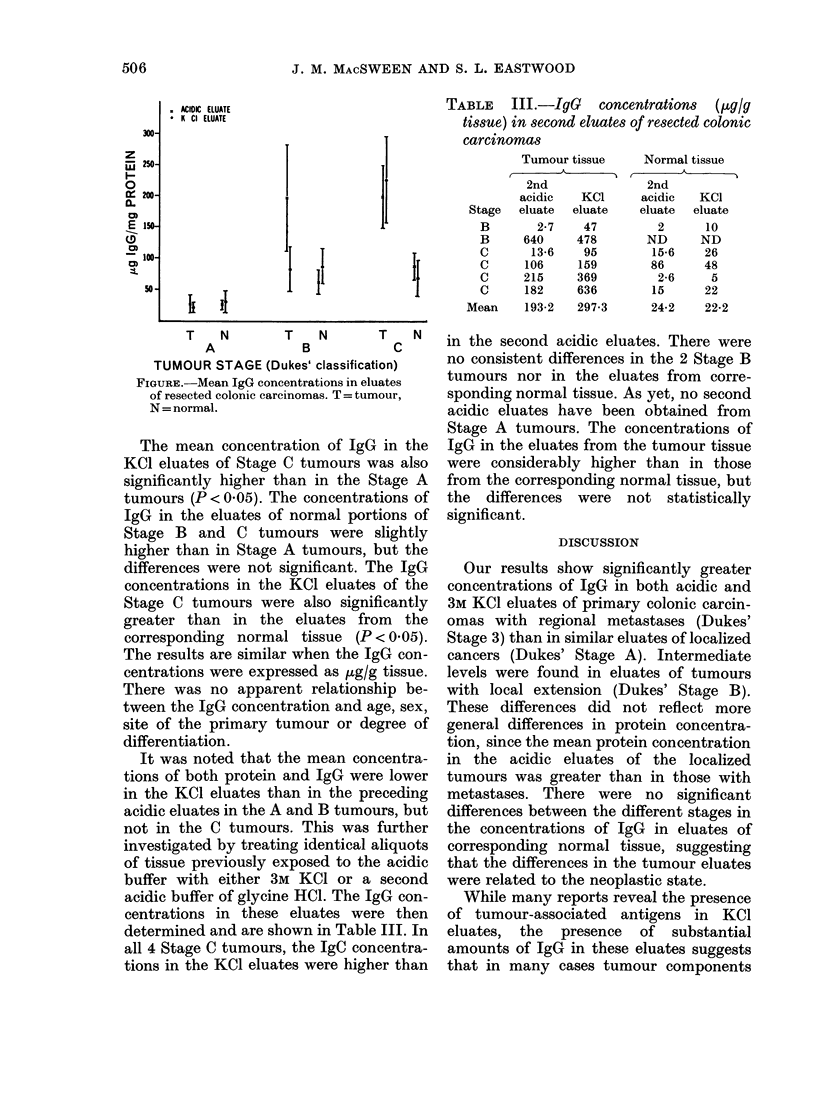

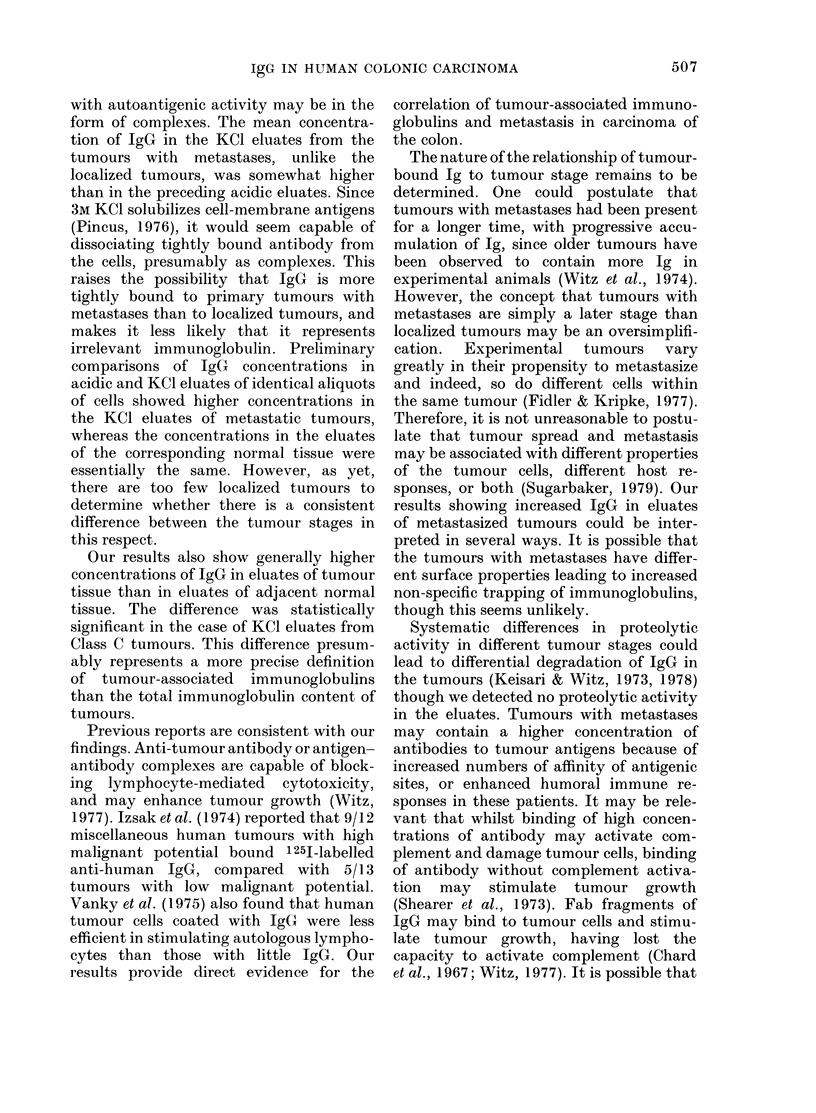

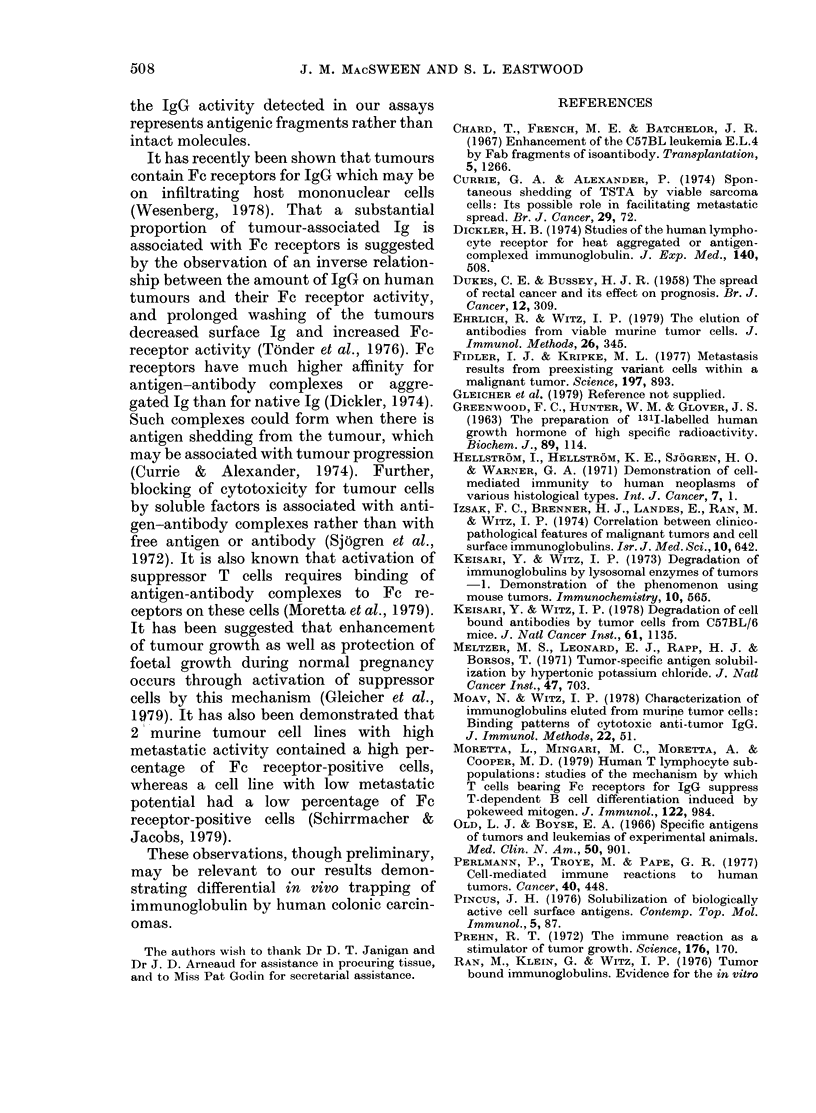

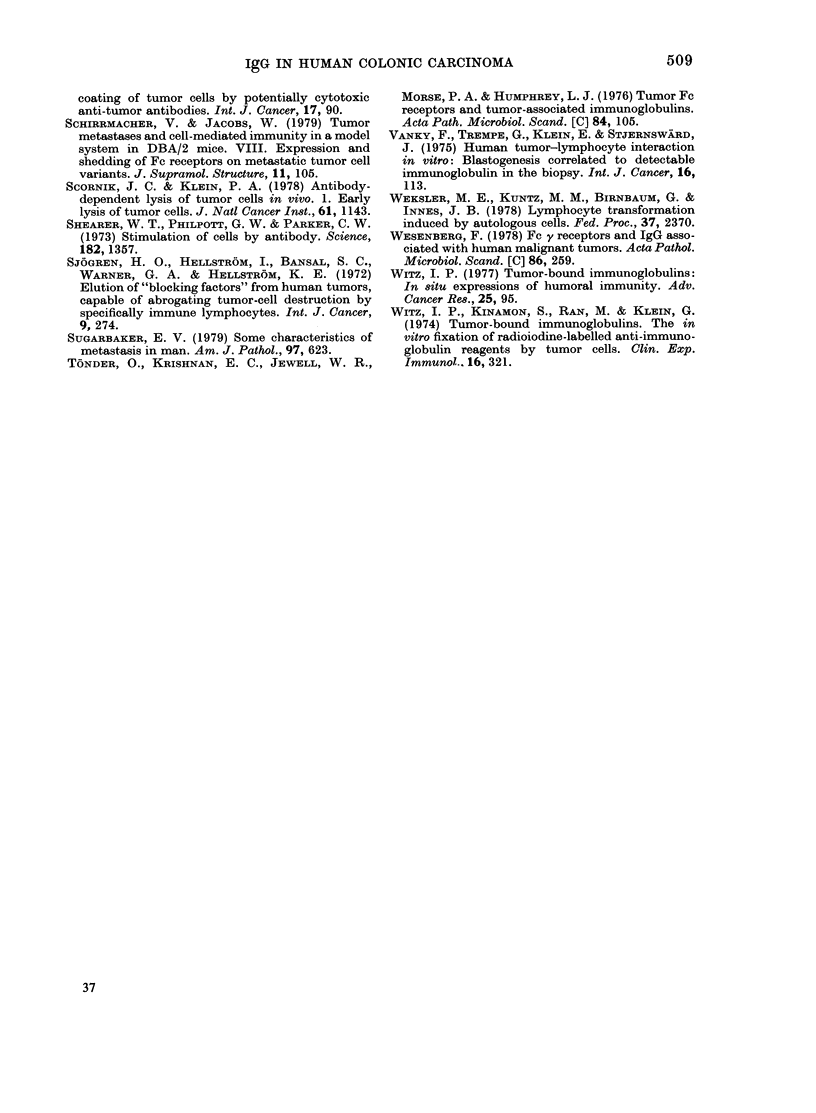


## References

[OCR_00891] Chard T., French M. E., Batchelor J. R. (1967). Enhancement of the C57BL leukemia E.L.4 by Fab fragments of isoantibody.. Transplantation.

[OCR_00897] Currie G. A., Alexander P. (1974). Spontaneous shedding of TSTA by viable sarcoma cells: its possible role in facilitating metastatic spread.. Br J Cancer.

[OCR_00909] DUKES C. E., BUSSEY H. J. (1958). The spread of rectal cancer and its effect on prognosis.. Br J Cancer.

[OCR_00903] Dickler H. B. (1974). Studies of the human lymphocyte receptor for heat-aggregated or antigen-complexed immunoglobulin.. J Exp Med.

[OCR_00914] Ehrlich R., Witz I. P. (1979). The elution of antibodies from viable murine tumor cells.. J Immunol Methods.

[OCR_00919] Fidler I. J., Kripke M. L. (1977). Metastasis results from preexisting variant cells within a malignant tumor.. Science.

[OCR_00926] GREENWOOD F. C., HUNTER W. M., GLOVER J. S. (1963). THE PREPARATION OF I-131-LABELLED HUMAN GROWTH HORMONE OF HIGH SPECIFIC RADIOACTIVITY.. Biochem J.

[OCR_00938] Izsak F. C., Brenner H. J., Landes E., Ran M., Witz I. P. (1974). Correlation between clinico-pathological features of malignant tumors and cell surface immunoglobulins.. Isr J Med Sci.

[OCR_00949] Keisari Y., Witz I. P. (1978). Degradation of cell-bound antibodies by tumor cells from C57BL/6 mice.. J Natl Cancer Inst.

[OCR_00943] Keisari Y., Witz I. P. (1973). Degradation of immunoglobulins by lysosomal enzymes of tumors. I. Demonstration of the phenomenon using mouse tumors.. Immunochemistry.

[OCR_00954] Meltzer M. S., Leonard E. J., Rapp H. J., Borsos T. (1971). Tumor-specific antigen solubilized by hypertonic potassium chloride.. J Natl Cancer Inst.

[OCR_00960] Moav N., Witz I. P. (1978). Characterization of immunoglobulins eluted from murine tumor cells: binding patterns of cytotoxic anti-tumor IgG.. J Immunol Methods.

[OCR_00966] Moretta L., Mingari M. C., Moretta A., Cooper M. D. (1979). Human T lymphocyte subpopulations: studies of the mechanism by which T cells bearing Fc receptors for IgG suppress T-dependent B cell differentiation induced by pokeweed mitogen.. J Immunol.

[OCR_00974] Old L. J., Boyse E. A. (1966). Specific antigens of tumors and leukemias of experimental animals.. Med Clin North Am.

[OCR_00979] Perlmann P., Troye M., Pape G. R. (1977). Cell-mediated immune reactions to human tumors.. Cancer.

[OCR_00984] Pincus J. H. (1976). Solubilization of biologically active cell surface antigens.. Contemp Top Mol Immunol.

[OCR_00989] Prehn R. T. (1972). The immune reaction as a stimulator of tumor growth.. Science.

[OCR_00993] Ran M., Klein G., Witz I. P. (1976). Tumor-bound immunoglobulins. Evidence for the in vivo coating of tumor cells by potentially cytotoxic anti-tumour antibodies.. Int J Cancer.

[OCR_01002] Schirrmacher V., Jacobs W. (1979). Tumor metastases and cell-mediated immunity in a model system in DBA/2 mice. VIII. Expression and shedding of Fc gamma receptors on metastatic tumor cell variants.. J Supramol Struct.

[OCR_01009] Scornik J. C., Klein P. A. (1978). Antibody-dependent lysis of tumor cells in vivo. I. Early lysis of tumor cells.. J Natl Cancer Inst.

[OCR_01013] Shearer W. T., Philpott G. W., Parker C. W. (1973). Stimulation of cells by antibody.. Science.

[OCR_01020] Sjögren H. O., Hellström I., Bansal S. C., Warner G. A., Hellström K. E. (1972). Elution of "blocking factors" from human tumors, capable of abrogating tumor-cell destruction by specifically immune lymphocytes.. Int J Cancer.

[OCR_01026] Sugarbaker E. V. (1979). Some characteristics of metastasis in man.. Am J Pathol.

[OCR_01030] Tönder O., Krishnan E. C., Jewell W. R., Morse P. A., Humphrey L. J. (1976). Tumor Fc receptors and tumor-associated immunoglobulins.. Acta Pathol Microbiol Scand C.

[OCR_01036] Vánky F., Trempe G., Klein E., Stjernswärd (1975). Human tumor--lymphocyte interaction in vitro: blastogenesis correlated to detectable immunoglobulin in the biopsy.. Int J Cancer.

[OCR_01043] Weksler M. E., Kuntz M. M., Birnbaum G., Innes J. B. (1978). Lymphocyte transformation induced by autologous cells.. Fed Proc.

[OCR_01049] Wesenberg F. (1978). Fc gamma receptors and IgG associated with human malignant tumours.. Acta Pathol Microbiol Scand C.

[OCR_01057] Witz I. P., Kinamon S., Ran M., Klein G. (1974). Tumour-bound immunoglobulins. The in vitro fixation of radioiodine-labelled anti-immunoglobulin reagents by tumour cells.. Clin Exp Immunol.

